# The Blossom Gang: co-producing research on FGM with second-generation young people in the UK

**DOI:** 10.1186/s40900-023-00457-y

**Published:** 2023-08-16

**Authors:** Saadye Ali

**Affiliations:** School of Health and Social Work, University of Hertfordshire, College Lane, Hatfield, AL10 9AB UK

**Keywords:** Participation, Youth, FGM, Second-generation, Community-based participatory research, Insider, Doctoral research

## Abstract

**Background:**

Female genital mutilation (FGM) is a practice that involves the removal of external female genitalia and is widely known as a violation of human rights. The custom is illegal in the United Kingdom (UK) and carries a sentence of up to 14 years in prison. This prohibition, along with the secretive nature of the practice, has led to limited research on the awareness of FGM on young people in the UK. Little is known about the process of involving young people in research about the topic.

**Methods:**

This paper is based on the findings of a Ph.D. project that used a community-based participatory research approach (CBPR). The research took a two-stage approach: stage one aimed to recruit nine young people aged 15–18, from the Southwest of England, who attended a 10-day training workshop to prepare them for stage two—data collection with young people aged 13–15. This paper focuses on the 10-day creative, collaborative workshops. The data collected from the collaborative workshops were analysed using thematic analysis.

**Results:**

Undertaking CBPR enhanced the quality and relevance of this research. Engaging young people as co-researchers was vital for the success of this project. By developing a collaborative learning environment, young people were able to build trusting relationships which flourished beyond the research project. In addition, the creative workshops enabled peer learning about FGM and inspired young people to learn new skills that was useful in their daily lives.

**Conclusion:**

The collaborative environment created in this project enabled an insightful learning experience for young people and researchers alike. Participants and facilitators formed relationships; participants learned new marketable skills and researchers gained new insights about FGM, from a young person’s perspective.

**Supplementary Information:**

The online version contains supplementary material available at 10.1186/s40900-023-00457-y.

## Abstract (plain language summary)

Making sure that young people are involved in research is important. It can sometimes be hard for young people to get involved with research involving a sensitive topic, such as female genital mutilation. This may be due to several reasons, one of which is the hidden nature of the practice, which may make it harder for researchers to engage and involve the youth. This project involved a group of second-generation youth from FGM-affected communities as co-researchers in a community based participatory research (CBPR) approach, which examined how second-generation youth, living in the United Kingdom (UK)—whether directly or indirectly affected by FGM—interpreted and understood the practice. We followed a two-stage process. Stage one involved recruiting and training young people to become co-researchers, and stage two involved the co-researchers conducting focus groups and interviews with their peers. The focus of this paper is stage one.

Nine young people aged 15–18, attended a 10-day creative workshop training programme [7] and worked with the researcher to develop participatory methods that would be used with young people aged 13–15 at stage two of the project. The workshops employed team-building approaches and interactive learning techniques, including drawing and writing. Participants and facilitators formed relationships; participants learned new marketable skills and researchers gained new insights about FGM, from a young person’s perspective. There were some difficult parts of running the sessions, such as, the time needed to make sure the sessions run smoothly.

**Supplementary Information:**

The online version contains supplementary material available at 10.1186/s40900-023-00457-y.

## Background

Female genital mutilation (FGM) is an overarching term used to define cultural practices that result in the modification of the female genitalia for social or cultural, rather than medical reasons [1]. The procedure has no known health benefits and often results in immediate and long-term health problems for those affected [2, 3].

The practice has been illegal in the United Kingdom since 1986, Prohibition of Female Circumcision Act [4]. This was superseded by the 2003 FGM Act, the scope of which was extended by the Serious Crime Act in 2015 [5]. These amendments add the offence of failure to protect a girl or women from FGM, provide lifelong anonymity for victims of FGM, and extend the scope of the law extra-territorially [6, 7].

The reasons behind the practice differ depending on the culture of which it is practiced [8, 9]; ranging from cultural to social and religious reasons, which include societal pressure, rites of passage, religious mandates, acculturation, and ideas of femininity, modesty, and sexuality [7, 10]. Due to these differences, a contextualised understanding of African immigrant beliefs and practices is particularly important in understanding what might be holding FGM in place, in a different, hostile, and illegal environment such as the UK.

Participation is one of the building principles of the United Nations Convention on the Rights of the Child [8]. More specifically, Article 12 of the Convention, enriches the right for children to participate in the decision-making process in issues that are of relevance to their lives, including decisions made in the private and public spheres. Furthermore, Goal 17: Target 5.2 and 5.2.2 of the United Nations Sustainable Development Goals [11], calls for the elimination of all forms of violence against women and girls as well as the reduction of the number of girls under the age of 15 subjected to sexual abuse. Researchers must, therefore, acknowledge young people as social players, who have the right to hold opinions and assume responsibility in society.

Young people are becoming increasingly involved in participatory research [12, 13]. Such research has sought to ‘empower’ young people not only by including their voices as experts but involving them throughout the research process by engaging them in meaningful and relevant ways, such that they avoid tokenism. This collaborative approach to involving young people recognises them as ‘experts in their own lives’ and offers researchers new insights and young people opportunities to learn new skills and knowledge, build their confidence and empower them to make positive changes in their lives [14–16]. Knowledge gained from participatory research is, therefore, culturally relevant and connected to the lives and experiences of young people. Consequently, findings are more likely to be translated into action than just knowledge generated purely for academic deliverables and which is not implemented [17].

The prohibition, along with the secretive nature of the practice, has led to limited research focusing on FGM with young people in the UK. Little is known about the process of involving young people in such a topic. The process, reflections and recommendations described in this paper are based on a doctoral research project which took a community based participatory research approach to examine how approaches aimed at preventing FGM can be improved and developed with second-generation young people in the UK [18, 19]. This paper draws on the findings of the training workshops, stage two, and sheds light on the training process and offers reflections from the author as an insider and researcher (Additional file 1).

### Project development

The rationale for this project was twofold. Due to the dearth in research exploring the views of FGM amongst second-generation youth and the sensitivity of the topic, it was necessary to explore such issues in a participatory and sensitive way. Secondly, as a registered nurse and an individual from an FGM at-risk community, I came across this custom at age 23, whilst working as a registered nurse in an acute setting and later whilst conducting research for my Masters' dissertation—it was during this time that I learnt that close family members were survivors of FGM.

There have been several debates surrounding the benefits and challenges of researchers conducting studies in familiar environments, particularly where they were born, raised, and belong. Dwyer and Buckle [20] argue that insider researchers are often able to engage research participants effectively and use their shared experiences to gather rich data. However, they may find it difficult to separate their personal experiences from those of the research participants [21, 22] and may also face issues of confidentiality, when interviewing members of their communities about sensitive topics [6, 22]. Outsider researchers are often valued due to their ability to remain objective and their emotional distance to the subjects, though they may find it challenging to gain access to research participants [23].

As a researcher, I held a level of understanding and respect for the group, and it was my background and positionality that enabled me to build trust, subsequently leading to successful recruitment. My positionality as an insider also enabled a positive level of engagement between young people. They were able to share their experiences and thoughts, which would otherwise be hidden to outsiders due to the topic under study. Furthermore, while I positioned myself as an insider, as the research progressed, I became much more aware of several aspects that would otherwise position me as an outsider, such as my professional background and age. Therefore, by utilising a community based participatory research approach and working closely with the communities, I occupied the ‘space in between’ [22] and was constantly aware of the changing nature of my positionality.

## Methods

A community based participatory approach (CBPR), was used in this study. Nine young people aged 15–18, attended a 10-day creative workshop training programme [7] and worked with the researcher to develop participatory methods that would be used with young people aged 13–15 at stage two of the project. The decision to focus on these age groups served two purposes. Firstly, recognising the limited existing research on young people's experiences of Female Genital Mutilation (FGM), this project aimed to contribute to the current scholarly literature by specifically exploring the viewpoints of second-generation youth. Secondly, as Relationship and Sex Education (RSE) is mandatory in UK secondary schools starting at age eleven, with guidance to address FGM, it was deemed appropriate to engage with students aged 13 and above. These students receive education on the physical and emotional harm caused by FGM, as well as the relevant UK laws surrounding this practice [23]. It was believed that this knowledge would enable young people to offer valuable insights and perspectives on the subject matter.

The workshops employed team-building approaches and interactive learning techniques, including drawing, and writing and addressed the following topics: sexual and reproductive health including awareness of FGM, safeguarding, intercultural communication, participatory methods, the study conceptual framework and epistemology, ethics and finally guidelines and exercises on conducting semi-structured interviews and focus groups in an empathetic and ethically sound way. The participatory nature of CBPR allowed for young people to engage by using a range of creative arts-based techniques, enabling flexibility in diverse settings. Creative techniques were applicable throughout the research process from identifying research questions, collecting, and analysing data, and interpreting as well as disseminating findings [23, 24]. Furthermore, using creativity offered opportunities for young people to draw on emotion and imagination [25, 26] and not rely solely on their verbal or written expression, which at times, may not be able to be conveyed through language [27]. Such approaches aided in exploring this complex topic, which may have been beyond the reach of words [28–30].

### Recruitment

The co-researchers were recruited through a community organisation in the Southwest of England, which works with BAME women from refugee backgrounds. The manager of the organisation was approached, and following meetings with the trustees agreed to assist in recruitment.

### The workshops

The training workshops commenced in October 2016, nine young people aged 15–18, and two facilitators gathered for a 10-day workshop, steered by the author, whose role was to keep time, facilitate sessions, manage arising issues, and ensure appropriate resources were available. It was important that we create a safe, trusting, collaborative environment to facilitate sharing and learning, generate ideas, develop skills, and achieve agreed goals. Workshop days were designed to maximise involvement, notably, to include less confident young people. Therefore, young people and facilitators engaged in group activities and played games lunched and spent breaks together. The workshops were conducted after school. Therefore, it was paramount we created room for activities and enabled a balanced atmosphere; this aided in maintaining momentum, energy, and interest for the co-researchers.

The creative workshops drew on the five principles of co-production, as highlighted by the National Institute for Health Research (NIHR) INVOLVE [31]. These are sharing power; including all perspectives and skills; respect and value the knowledge of all—everyone is of equal importance, and reciprocity—everybody benefits from working together, as well as building and maintaining relationships.

### Ethics approval and consent to participate

The Research Ethics Committee of the Faculty of Health and Applied Science at the University of the West of England, Bristol, UK granted ethical approval for this research in August 2016 (reference: HAS.16.07.176). This research adhered to the British Educational Research Association ethical guidelines [32].

#### Consent and confidentiality

All young people involved in this project provided written, informed consent to take part. Due to the nature of the research topic, written consent was also obtained from parents for all young people, and this included audio recording all sessions. Furthermore, a confidentiality agreement, covering safeguarding, was agreed with all co-researchers. Young people were also encouraged to use pseudonyms in all discussions.

In our group, conversations also included discussing confidentiality and disclosures. In reality, ‘a conversation’ on confidentiality and disclosures, became a central theme that was periodically revisited and redefined throughout the training. An example of these discussions is given below, followed by the norms the group agreed upon.

Facilitator: “So, we wanted to have a conversation with you all around some thoughts and ideas of what you might suggest for ways that we can ensure that this is a safe space for everyone, Is that OK with everyone?”

Furthermore, the emotional health and wellbeing of young people was important for everyone involved. The topics under discussion may have acted as triggers, especially if the young people had undergone FGM. We, therefore, sought support from a safeguarding lead as well as the organisation we had recruited from, who support women and girls in these issues. Young people were encouraged to contact either the safeguarding lead or the gatekeepers if any issues arose. Additionally, all the facilitators were vigilant, sensitive, and supportive. The author held a level 3 safeguarding certificate. We also ensured that at the end of each day, the researcher and participatory consultant held debrief sessions to discuss concerns that had arisen during the workshop.

To enable and facilitate a supportive session, we adopted a range of creative techniques, combined with activities and exercises, and built breaks into the sessions. Boyden and Annew [33] emphasise the importance of using warm-ups and cool-downs to create a participatory atmosphere and improve group solidarity. However, the authors also warn that these activities may present problems, unless facilitators ensure they are handled accordingly, stating that warm-ups should: be non-threatening, be cultural, religious, and gender-appropriate, non-competitive, and challenge the power imbalance, inclusive and within the physical capabilities of all participants [34].

The rationale for these activities was to break down tensions and foster an open, participatory environment. We started with a game called the ‘teen talk jar’, to aid in stimulating conversations. Each person was given a question and were encouraged to answer, for example:

If you could give up TV for one year, what would you do with your time?

Some answers included:

“I would talk to my siblings probably, annoy my brothers more.”

“I would read, I don’t watch TV anyway, too much schoolwork.”

It was important to encourage participation and enable the process of co-learning; therefore, both co-researcher and facilitators engaged in the discussions. Researcher involvement can aid co-researchers to feel more comfortable in sharing information and close the hierarchical gap between researcher and co-researcher that traditional research encourages [34–36], thus promoting dialogue rather than an interrogation. Following introductions and the icebreakers, the co-researchers were encouraged to choose ground rules, thus also establishing group norms. Johnson and Johnson [37] stated that norms are rules established by groups to regulate behaviours of all members, adding that norms cannot be imposed in a group. Instead, they are formed through a process of interaction among members.

### Safeguarding

Safeguarding was a critical consideration throughout the research conducted in this sensitive area. The concept of "safeguarding" extends beyond child protection to encompass preventive measures. This entailed the necessity of averting any potential harm to vulnerable individuals, which was particularly relevant in the context of potential disclosures of FGM risk during the research.

While participants were not explicitly asked to share personal experiences of FGM, the nature of the project raised the possibility that some participants may have had personal experiences with FGM or been at risk of it. To ensure utmost safety, rigorous safeguarding protocols were followed. This involved close collaboration with organizations and agencies dedicated to supporting and advising young people within Black, Asian, and Minority Ethnic (BAME) communities affected by FGM. The project benefited from the assistance of a Children's Safeguarding Lead from the Southwest Council, who served as a point of referral if needed.

Furthermore, the principal researcher, a registered nurse with a Level 3 Safeguarding qualification, was present throughout all stages of data collection to promptly address any potential safeguarding concerns that may have arisen. These measures were implemented to prioritise the welfare and well-being of the participants, ensuring their safety, and providing appropriate support when necessary.

### Participatory decision making

Displaying trustworthiness and gaining trust are essential components in CBPR research. This is an ongoing process which must be earned and maintained [38]. After the introductory session and the training on FGM, the co-researchers began to bond. They met at college and walked to training together and discussed other issues that were relevant to them; for example, in between activities, the co-researchers discussed their school activities and exams:

Zuli: “I was so ill; I didn’t do any revision. I was like, all this mock, I don’t really care about the mock. But the teacher is really good.”

Rwaida: “She is, I know. I managed to get 68 per cent in one of the mocks.”

Although these two girls went to the same school, they had not spoken to each other until they formally met in training. These conversations illustrate the groups forming and perhaps bonding, due to similarities in their experiences, such as their lifestyle characteristics, shared beliefs, interests, and religious backgrounds [37].

Possibly the most critical aspect of the sessions was the commitment shown by the co-researchers. For instance, on one occasion, even though a co-researcher was celebrating her birthday, she had decided to attend the training, which was after school. The other co-researchers and facilitators decided to celebrate with her on the day, and this is when we could see a sense of kinship develop.

Rwaida: “Looks yummy, what type of cake is it? Does this have actual carrots in? I didn’t know that carrots go in cakes.”

Zuli: “Yes, it’s carrot cake.”

Rwaida: “It’s actually got carrots [Rah!]”

The group developed a sense of togetherness, and this was important, because an active group refers to mutual recognition among members as well as having a sense of belonging to the group [21]. This belonging can be in the form of shared social norms, values, and a sense of shared purpose, nurturing a sense of membership. In relation to this research, the group was composed of nine young people, a small number, which may have made forming relationships easier.

In addition to forming relationships, Brown and Lohr [39] argue that young people may identify with groups to develop a sense of identity. Therefore, group names that young people give themselves illustrate shared beliefs and interests. As such, the co-researchers discussed amongst themselves, and named their group—the ‘Blossom Gang’. When exploring what this title meant to them, young people articulated a range of meaningful connotations:

“Blossom symbolises flowers, growth, nature, and development. This links to Female genital mutilation (FGM), sexual health and females’ relationships. Firstly, because females’ private parts are often referred to as flowers, secondly, sexual health awareness requires growth and development of peoples understanding…and lastly; relationships are about growth and feelings blossoming. On the other hand, while ‘Gang is usually applied in a negative context, it can be used to represent youthfulness and informal relationships, it can be used to symbolise unity and togetherness.”

### Reflections and learning

The five fundamental principles for co-production identified earlier [26] provide a useful critical framework for reflection from the researcher’s point of view and have been used to form the discussion on this section.

#### Sharing power

Due to the nature of the project and it being part of a Doctoral study, it was challenging to hand over complete responsibility to the co-researcher, this was due to the academic deliverables imposed upon the study. This sentiment is shared by NIHR [31], by which they acknowledge that sharing power does not mean involving everyone at every decision made, there are roles which people take and cannot be shared. Therefore, the idea to study FGM did not originate from the co-researchers, as this had to be submitted as part of a milestone for the PhD before ethics approval. However, following ethics and consent, the researcher engaged with the co-researchers in all decisions made, they reviewed the proposed interview and focus group agenda and generated new ideas to be included, also decided which participatory methods would be used. As such, the decision-making process was collaborative, with shared responsibility and understanding.

#### Include all perspectives and skills

In this project, the aim was to include young people from diverse backgrounds. Therefore, the co-researchers recruited were ethnically diverse. Although only one of the co-researchers was male, he engaged with the training equitably. The training deliverables and output, therefore, reflected on this group of young people, and may not be representative to all second-generation young people from FGM affected communities in the UK.

The 10-day workshops may have also been a barrier for some to participate, as not all were able to attend the specified sessions. The researcher ensured that an arrangement was made for those who missed a particular session to catch up. This flexibility ensured inclusivity. However, flexibility is expressive and time-consuming, mainly due to time restrictions of completing the thesis within 3 years. Furthermore, by involving parents and gaining their consent and due to FGM being illegal in the UK, this meant that some young people who may have undergone FGM or at risk of it might not have been recruited as parents may have been less keen to engage, due to the fear of being caught.

#### Respect and value the knowledge of all—everyone is of equal importance

Although the research did not intend to ask young people if they hand undergone the practice, we were aware that they might have been survivors. Therefore, this might have made it difficult for them to join group discussions. The co-researchers were offered a range of options, both audio and visual, that would enable them to contribute. Co-researchers were encouraged to use their skills in the sessions, for example, whilst some expressed themselves through drawing, others used poetry. The knowledge created through poetry, drawing and writing was, therefore, of equal value.

#### Reciprocity—everybody benefits from working together

This creative, informal, collaboration offered co-researchers an opportunity to focus on their strengths but also, enable peer engagement and learning. The young people were brimming with ideas and insights which they were eager to communicate. The co-researchers felt that there were specific concerns that young women from Black Asian Minority Ethnic (BAME) groups faced:

Uba: “Not fitting in, not being accepted… being different. I think it depends on where you grow up. For example, if I live in Easton and go to school in Easton, I would be more accepted as opposed to living in Easton but going to like Colston.”

Suraya: “I don’t think it’s just about where you live, though. I feel like, it is most of a societal issue you could be accepted in your community… but like if you apply for a job and they see your name is different, they are not going to really invite you.”

Dolla Sign: “We were talking about how your hair affects the jobs you get as well. You are often forced to have your hair slicked back or in braids, not an afro, so that you did ‘look’ a certain way.”

These conversations shed light on important issues they faced in their lives. Their discussions about topics like "racial profiling, mental health, money issues," among others, highlighted their experiences as minorities in a European society. These conversations signify the effectiveness of the Community Based Participatory Research approach (CBPR) in fostering open dialogue.

Additionally, it is essential to delve deeper into the conversations initiated by the co-researchers regarding Female Genital Mutilation (FGM) within the broader context of race and belonging. Exploring how perceptions of FGM influence the way migrant groups are perceived by their host societies can provide valuable insights into the experiences of these communities in Europe. By examining the intersection of FGM, race, and societal integration, we can better understand the complexities faced by migrants affected by FGM and how it impacts their interactions with the host community.

By reframing the discussion to emphasise the organic conversations that emerged from the co-researchers' experiences, we can gain a more comprehensive understanding of the challenges and dynamics surrounding FGM within migrant or minority groups in Europe. This approach allows us to acknowledge the interconnectedness of FGM with broader issues of race, identity, and belonging, and consider its implications on social perceptions and integration within the host society.

Having young people share their experiences, the researcher was also able to learn and gain insights into issues that may not always been outlined amongst adults. The young people also acknowledged the learning opportunities that the workshops offered,

“… I think in the first few sessions, we had quite a lot of ice breakers, and we had the chance to get to know each other as well. We had the activity where we got to know each other, compare what we had in common, and what we didn’t. Just getting to know everyone and getting comfortable, cause obviously, we would be speaking about quite intimate topics and things that people might not be comfortable to talk about… but being able to know each other and being comfortable in that private space made it a lot easier to actually settle us and get our voices out.”

What is evident in this extract is the relationship formed amongst the young people; they were then able to share knowledge and learn on issues that may otherwise be difficult to approach. The adult researcher’s role was to facilitate, guide, support and respect the young people’s skills and experiences to hand power over and acknowledge them as equal members of the research team.

#### Build and maintain relationships

The ability to build and maintain relationships was the building block for this project. Trusting relationships, build on mutual respect are the heart of power-sharing, decision making and learning. The project enabled young people from diverse backgrounds to meet and learn. These young people joined having an established identity and distinct sets of skills, experiences and expertise, the research team had worked together before the inception of this project and bought insider knowledge and insights from their experiences, but also acknowledge the importance to enabling young people to achieve their aims.

### Impact: beyond the training

Central to the entire discipline of CBPR is the concept of impact [39]. Having discussed and evaluated the training, the co-researchers and facilitators engaged in a discussion regarding the usefulness of the training in their everyday lives. Through active engagement, communities should become more empowered and better equipped to make long-term personal and social change. The question of ‘who benefits’ from the training and research is of relevance in co-production. Having worked with community organisations, the researcher would often hear their frustrations of feeling ‘used by universities’, where their names would be included in funding applications to obtain grants but, once that was successful, they would have limited involvement in projects. We wanted to ensure that young people gained skills that they could use in their future work.

It was interesting to hear that the skills they had learned were being transferred to college, as Dolla Sign, comments:

“At school, cause I do health and social care, I do get asked questions like how would you interact with other people if certain things would happen in your community, stuff like that would come up, and I’d know how to answer it… recently we did FGM, and they were giving information to others, and I was able to give extended information to them because they didn’t know as much as I do now, so that helped.”

Zuli mentions the skills she used during her summer job. Although she had worked with young people before, this training provided a level of confidence.

“For my summer job, I have to talk to young people, and even though I have been doing it before, I wasn’t as confident, and I couldn’t really… I struggled to communicate with young people effectively. Like we had to teach them leadership skills, this research allowed me the confidence to do so… I thought about planning sessions and what sort of things you could do for different learners. For example, I like, prefer presentations, but when talking to young people, I am not just going to use presentations. I thought about other ways to learn, and I thought this project really helped me in that. I am a lot more confident in public speaking and doing presentations. So, it’s not just helped during the research but outside as well.”

It appears that not only did the co-researchers learn new and marketable skills, but they were also able to utilise them in their jobs.

## Discussion

This paper contributed to the wider research by serving as an exemplar on how to conduct a community-based participatory research study with young people within a doctoral research journey. The findings of this project shed new knowledge to FGM research, more novel is the engagement of second-generation young people in the UK. To the best of our knowledge, this is the first research of its kind. The results highlight that using CBPR improved and contributed to study recruitment as well as the participation of young people. This approach also had an impact on research quality and relevance due to the involvement of young people in issues that affect them. However, it was apparent that FGM may not be the only issue of concern for young people of the second generation living in the United Kingdom.

Birch and Miller [40] draw upon Walker’s [41] ethics of responsibility to examine the tension between PhD requirements and CBPR, highlighting the sense of responsibility needed to sustain engagement with co-researchers throughout the research. They also identify the impact of time pressures, funding requirements, professional interests, and academic regulations on the process of discontinuing contact with co-researchers in the final stages. Whilst Bradbury [42] suggest that the overarching purpose of CBPR is defined by its engagement with issues of pressing concern to certain people, where members of marginalised groups would ideally initiate projects of this kind. This aspect of participation presents challenges for doctoral projects. PhD researchers are required to meet certain milestones: for this project, for instance, the researcher was awarded a studentship, and a broad proposal had been developed before the commencement of the study. In addition to this, there was a requirement to submit a further, more refined proposal within 3 months of the research start date. This creates a fundamental challenge for the CBPR methodology, in which researchers are not supposed to pre-empt how projects will unfold [43, 44].

### Strengths and limitations

The linear process was challenging during the early stages of this study. Although I am from an FGM-affected community, and a second-generation immigrant, it felt inappropriate to write the proposal and the ethics application without prior consultation with the young people who would take part in the research. Ultimately, there were several reasons why this approach did not jeopardise the study. Herr and Anderson [44] emphasise that social sciences research is often emergent in design. Therefore, it was permissible to build uncertainty into research proposals and ethics applications. Furthermore, once the ethics application had been accepted, and co-researchers recruited, we met and discussed the project’s aims and objectives, space was given to reword or amend them accordingly. In hindsight, although the use of CBPR presented challenges, unique opportunities also arose whilst collaborating with young people.

## Conclusion

This paper outlines the process by which the training was delivered, and the lessons learnt. The workshop approach, rooted in creative participatory approaches, transformed the project from a one-sided approach, to one that explored female genital mutilation from a young person’s perspective. Approaches that aim to work with marginalised groups require thorough planning, commitment, resources and most importantly—time—which should not be underestimated. However, when conducted appropriately, CBPR holds a transformative capacity to empower vulnerable people to influence policy, services as well as decisions which affect their lives.

### Supplementary Information

**Additional file 1**. GRIPP2 (Guidance for Reporting Involvement of Patients and the Public) Framework Overview.

## Data Availability

Due to privacy restrictions, the data sets generated and analysed in the present study are not publicly available, as they contain information that may compromise the participants' anonymity.

## Abbreviations

FGM: Female genital mutilation
CBPR: Community-based participatory research
BAME: Black, Asian, and ethnic minority
