# Supplementary material for: The Blossom Gang: co-producing research on FGM with second-generation young people in the UK
Source: Res Involv Engagem. 2023 Aug 16;9:68. doi: 10.1186/s40900-023-00457-y (PMC10428588; doi:10.1186/s40900-023-00457-y)
Supplement: Supplementary file 1 — Additional file 1. GRIPP2 (Guidance for Reporting Involvement of Patients and the Public) Framework Overview. [file 40900_2023_457_MOESM1_ESM.docx]

**Additional file 1: GRIPP2 short form**

| Section and topic | Item | page No |
| --- | --- | --- |
| 1: Aim | **Report the aim of PPI in the study**: The process, reflections and recommendations described in this paper are based on a doctoral research project which took a community based participatory research approach to examine how approaches aimed at preventing FGM can be improved and developed with second-generation young people in the UK. The aim of this paper is to draw on the findings from the training workshops and shed light on the training process. The paper also offers reflections from the author as an insider and researcher. | p. 4 |
| 2: Methods | **Provide a clear description of methods used for PPI in the study**: Nine young people aged 15-18, attended a ten-day creative workshop training programme and worked with the researcher to develop participatory methods that would be used with young people aged 13-15 at stage two of the project. The workshop employed team-building approaches and interactive learning techniques, including drawing, and writing and addressed the following topics: sexual and reproductive health including awareness of FGM, safeguarding, intercultural communication, participatory methods, the study conceptual framework and epistemology, ethics and finally guidelines and exercises on conducting semi-structured interviews and focus groups in an empathetic and ethically sound way. | p. 6-7 |
| 3: Study Results | **Outcomes—Report the results of PPI in the study, including both positive and negative outcomes:** Undertaking CBPR enhanced the quality and relevance of this research. Engaging young people as co-researchers within the research process was vital to the success of this project. By developing a collaborative learning environment, young people reported able to build trusting relationships, which flourished beyond the research project. Furthermore, the creative workshops enabled peer learning about FGM; the young people reported having learnt new skills that were useful in their daily lives. Besides, both young people and researcher were positive on collaborations with more difficult populations like young people in sensitive topics (see Result section of publication for a more detailed description of the process with young people and researcher). | p. 17 |
| 4: Discussion and conclusions | **Outcomes—Comment on the extent to which PPI influenced the study overall. Describe positive and negative effects:**  Patient and researcher involvement at the conception of this study helped in gaining a sense of an equal dialogue. Ideas and doubts about the scope were discussed with researchers as well as young people to test whether this research would indeed be relevant. The ability to build and maintain relationships was the building block for this project. Trusting relationships, build on mutual respect are the heart of power-sharing, decision making and learning. The project enabled young people from diverse backgrounds to meet and learn. These young people joined having an established identity and distinct sets of skills, experiences and expertise, the research team had worked together before the inception of this project and bought insider knowledge and insights from their experiences, but also acknowledge the importance to enabling young people to achieve their aims. | p. 11-16 |
| 5: Reflections/critical perspective | **Comment critically on the study, reflecting on the things that went well and those that did not, so others can learn from this experience**:  Due to the nature of the project and it being part of a Doctoral study, it was challenging to hand over complete responsibility to the co-researcher, this was due to the academic deliverables imposed upon the study. However, following ethics and consent, the researcher engaged with the co-researchers in all decisions made, they reviewed the proposed interview and focus group agenda and generated new ideas to be included, also decided which participatory methods would be used. As such, the decision-making process was collaborative, with shared responsibility and understanding. Despite this, the study adds relevant knowledge on youth-involvement in FGM research and gives insights in possibilities to involve challenging populations. The study provides a helpful tool for researchers to start collaborating in their projects in the future. | p. 11 |
